# Functional vertical connectivity of microbial communities in the ocean

**DOI:** 10.1126/sciadv.adj8184

**Published:** 2024-05-23

**Authors:** Shi Chen, Zhang-Xian Xie, Ke-Qiang Yan, Jian-Wei Chen, Dong-Xu Li, Peng-Fei Wu, Ling Peng, Lin Lin, Chun-Ming Dong, Zihao Zhao, Guang-Yi Fan, Si-Qi Liu, Gerhard J. Herndl, Da-Zhi Wang

**Affiliations:** ^1^ State Key Laboratory of Marine Environmental Science/College of the Environment and Ecology, Xiamen University, Xiamen 361005, China.; ^2^ Southern Marine Science and Engineering Guangdong Laboratory (Zhuhai), Zhuhai 519082, China.; ^3^ School of Resource and Environmental Sciences, Quanzhou Normal University, Quanzhou 362000, China.; ^4^ BGI-Shenzhen, Shenzhen 518083, China.; ^5^ College of Life Sciences, University of Chinese Academy of Sciences, Beijing 100049, China.; ^6^ Qingdao Key Laboratory of Marine Genomics, BGI-Qingdao, BGI-Shenzhen, Qingdao 266555, China.; ^7^ Qingdao-Europe Advanced Institute for Life Sciences, BGI-Shenzhen, Qingdao 266555, China.; ^8^ Key Laboratory of Marine Genetic Resources, Third Institute of Oceanography, Ministry of Natural Resources, No. 184, Daxue Road, Siming District, Xiamen 361005, Fujian, China.; ^9^ Department of Functional and Evolutionary Ecology, Bio-Oceanography and Marine Biology Unit, University of Vienna, Djerassiplatz 1, 1030 Vienna, Austria.; ^10^ NIOZ, Department of Marine Microbiology and Biogeochemistry, Royal Netherlands Institute for Sea Research, Utrecht University, 1790 AB Den Burg, Texel, Netherlands.

## Abstract

Sinking particles are a critical conduit for the transport of surface microbes to the ocean’s interior. Vertical connectivity of phylogenetic composition has been shown; however, the functional vertical connectivity of microbial communities has not yet been explored in detail. We investigated protein and taxa profiles of both free-living and particle-attached microbial communities from the surface to 3000 m depth using a combined metaproteomic and 16*S* rRNA amplicon sequencing approach. A clear compositional and functional vertical connectivity of microbial communities was observed throughout the water column with Oceanospirillales, Alteromonadales, and Rhodobacterales as key taxa. The surface-derived particle-associated microbes increased the expression of proteins involved in basic metabolism, organic matter processing, and environmental stress response in deep waters. This study highlights the functional vertical connectivity between surface and deep-sea microbial communities via sinking particles and reveals that a considerable proportion of the deep-sea microbes might originate from surface waters and have a major impact on the biogeochemical cycles in the deep sea.

## Teaser

Functional vertical connectivity of microbes through the oceanic water column importantly contributes to the deep-sea communities.

## INTRODUCTION

Phytoplankton produce organic matter in the sunlit surface ocean, and up to 40% of this photosynthetically generated organic matter is exported to the deep ocean as detrital particulate organic matter (POM) in the form of decaying phytoplankton and fecal pellets generated by zooplankton grazing (*1*–*4*). The flux of sinking particles reaching the deep-sea realm (defined here as depths below 200 m) is to a large extent controlled by the attached prokaryotes through a series of activities, e.g., aggregation/disaggregation, POM degradation, and trophic transfer (*5*–*7*). Sinking particles are the major carbon and energy source for the deep-sea microbiota (*8*) and are also heavily colonized by surface microbes, which are transferred from the euphotic layer to the deep sea (*9*). Therefore, most of the dominant prokaryotic groups in the deep sea are also detected in the particulate fraction of euphotic waters (*9*–*12*), indicating that sinking particles potentially serve as vectors for viable particle-attached (PA) microbes from surface waters into the deep sea. Thus, these surface-derived microbes have a profound impact on the structure and biogeography of deep-sea microbial communities (*9*).

16*S* ribosomal RNA (rRNA)–based studies of size-fractionated microbial communities along the oceanic water column revealed a strong particle-mediated microbial connectivity between the surface and deep ocean (*9*, *12*, *13*). However, we have only rudimentary knowledge on the extent that the particle-mediated export of surface microbes into the deep sea is determining the functioning of the deep-sea microbial communities. Especially, whether and if so, how the surface water–derived microbes adjust their metabolic behavior to the marked environmental change during the descent to the ocean’s interior has rarely been investigated. From the surface to the deep sea, physical and chemical factors such as light, temperature, pressure, dissolved oxygen, and nutrients exhibit notable depth gradients. In contrast to the sunlit surface ocean characterized by phytoplankton production, the deep-sea realm is characterized by darkness, high hydrostatic pressure, low temperature, and low bioavailability of organic substrate for heterotrophic microbes (*3*, *14*, *15*). This implies that surface microbes attached to sinking particles are faced with increasing survival challenges when they are transported to the deep sea (*16*). It has been hypothesized that marine microbes regulate their physiological metabolisms in a complex pattern by developing comprehensive adaption mechanisms to survive in changing environments (*17*, *18*). Dynamic marine environments shape diverse bacterial stress response systems, which enable bacteria to adapt to various environmental changes such as temperature, pH, pressure, oxidative stress, and nutrient availability (*19*–*21*). Moreover, the surface microbes transferred to the deep ocean are faced with an increasingly recalcitrant organic matter pool. Studies show that bathypelagic microbes can regulate their metabolic strategies to enhance the utilization of POM as carbon and energy source in the deep sea (*22*). However, direct evidence on the microbial response mechanisms to the marked environmental changes during the transfer from the surface to the deep sea is still lacking.

Here, we investigate how PA microbes adjust their functional behavior to respond to the marked environmental changes during the transfer from the surface to the deep sea and test the hypothesis that surface microbes have the potential to survive in the deep-sea environment. We examined the protein and taxa profiles of both free-living (FL; 0.2 to 1.6 μm) and PA (1.6 to 200 μm) microbial communities collected from the surface waters down to 3000 m depth at two stations, the SouthEast Asian Time-series Study (SEATS) and Southern South China Sea (SS1) characterized by very different hydrographic conditions in the oligotrophic South China Sea (fig. S1 and table S1) using a combined metaproteomic and 16*S* rRNA amplicon sequencing approach. Specifically, we tested the hypothesis that surface-derived microbes contribute a large proportion of the functioning of deep ocean microbial communities. We show both taxonomic and functional vertical connectivity between surface and deep-sea microbial communities and reveal unique response mechanisms of the surface-derived microbes to the marked environmental changes during the particle-mediated transfer through the water column allowing them to survive in the bathypelagic ocean and ultimately, contributing to a considerable extent to the functioning of deep-sea microbial communities.

## RESULTS

### Vertical connectivity of microbes in the water column

Most of the amplicon sequence variant (ASV)– and protein-based microbial communities from the surface layer (5 m) were clustered together regardless of the stations, indicating depth-structured communities (fig. S2). The observed richness of the PA fraction increased with depth reaching the highest value at 3000 m depth. In contrast, the observed richness of the FL fraction reached its highest value at 200 m depth and then decreased toward greater depth (fig. S3). The total (FL + PA) observed richness exhibited a similar trend as the PA fraction (fig. S3). The microbial communities at 750 and 3000 m depths were much closer related to the surface microbial communities (5 m) in the PA fractions than in the FL fractions at both stations (fig. S4), indicating a link between surface and deep-sea microbial communities, especially for the PA fractions.

**Fig. 1.**
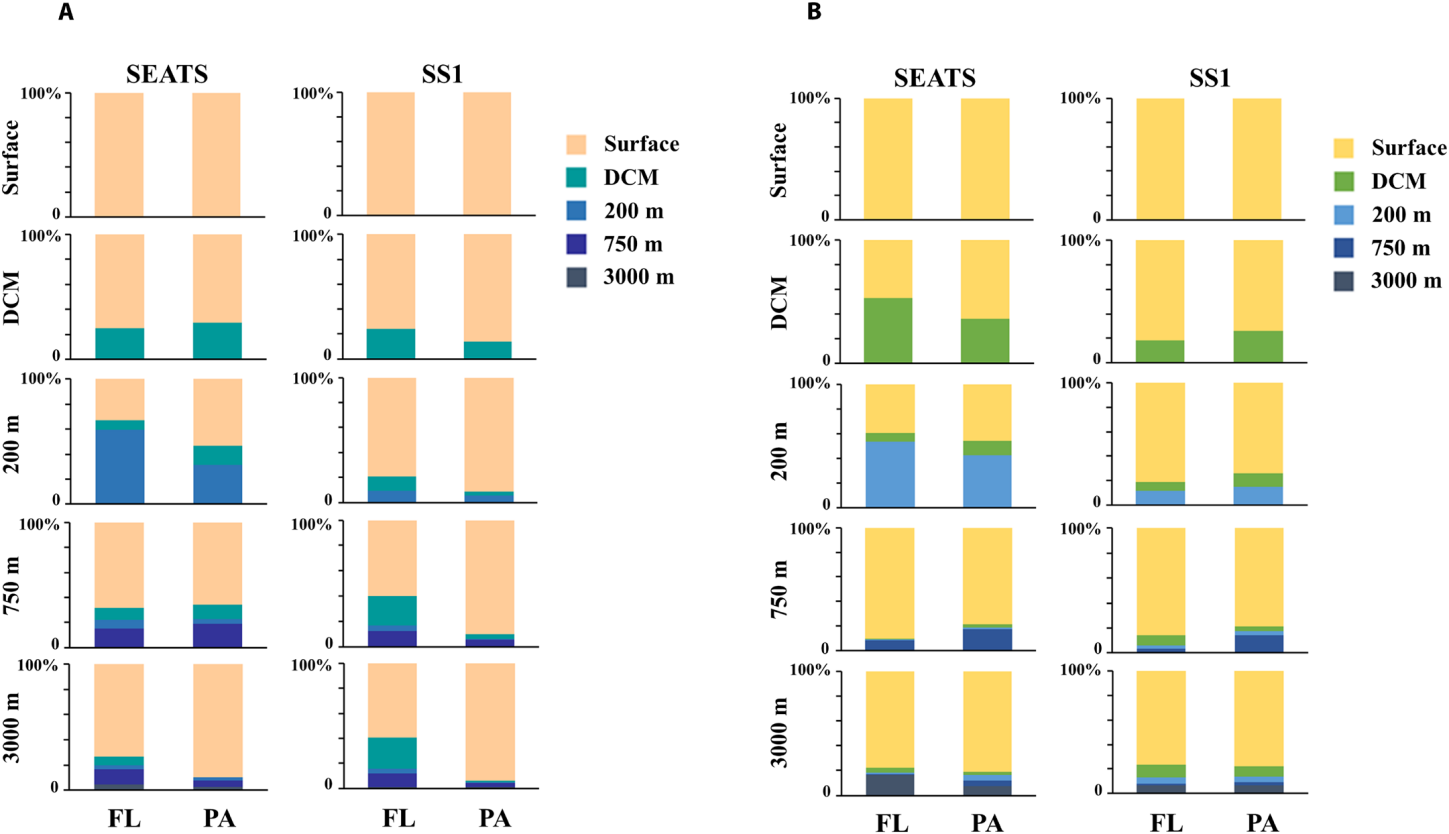
Contribution of the ASVs and proteins categorized as surface, DCM, 200, 750, and 3000 m in each size fraction (FL, 0.2 to 1.6 μm; PA, 1.6 to 200 μm) at stations SEATS and SS1. The contributions are expressed as the relative abundance of ASVs (**A**) and proteins (**B**) by percentage. The category of each ASV or protein is defined as the depth where it is first detected in any size fraction, considering a directionality from surface to bathypelagic waters.

To investigate the taxonomic and functional vertical connectivity between microbial communities from surface waters to the deep sea, we further examined whether ASVs and proteins present in one depth layer could also be detected in other layers of the water column (Fig. 1). Following a method previously described (*9*), the ASVs and proteins were categorized into five groups: The surface (5 m) and deep chlorophyll maximum (DCM; 75 m at SEATS and 100 m at SS1) layer in the euphotic zone, 200 and 750 m in the mesopelagic zone and 3000 m in the bathypelagic zone. The depth-related categories of ASVs and proteins were defined by the depth where they were first detected assuming a directionality from surface to bathypelagic waters based on the in situ hydrographic conditions (fig. S1). Although a few shared ASVs or proteins were observed between PA and FL fractions at the same water layer, indicating potential transformation of material between the two fractions via aggregation or detachment (fig. S5), this did not affect the definition of the depth-related categories of the ASVs or proteins due to the consideration of both fractions. That is, when an ASV or a protein was detected in a certain water layer, it should not be detected in any fractions from the upper layers. On the basis of this definition, even though new ASVs appeared continuously from one depth to a deeper layer, microbial communities from all depths and the two size fractions were largely dominated by the ASVs that were also present in surface waters (“surface” ASVs) (Fig. 1A). Surface ASVs contributed 73% to the ASV abundance in waters below 200 m and up to 79% in the bathypelagic layer (3000 m), indicating a large contribution of surface microbes to deep-sea microbial communities at the compositional level. At both stations, SEATS and SS1, the variation in the source of proteins in the different water layers (Fig. 1B) was similar to that of the ASVs (Fig. 1A), indicating a tight coupling between the taxonomic structure and the functional profile of microbial communities transferred via sinking particles into the deep waters. Although new proteins appeared continuously from one depth to the deeper layer, protein profiles of microbial communities from all depths and the two size fractions were largely dominated by proteins present in surface waters (surface proteins) (Fig. 1B). Surface proteins contributed 72% to the protein abundance in deep waters and up to 78% in the bathypelagic layer, demonstrating a considerable contribution of surface microbes to the functioning of deep-sea microbial communities. Moreover, the average contributions of surface ASVs to the deep-water ASVs at the SEATS and SS1 stations were 66 and 79%, respectively, while the contributions of surface proteins were 65 and 79%, respectively, indicating a stronger compositional (χ = 4.24 and *P* = 0.04) and functional (χ = 4.86 and *P* = 0.03) vertical connectivity at the SS1 station than at the SEATS station (Fig. 1).

### Particles as vectors for vertical connectivity of microbial communities

**Fig. 2.**
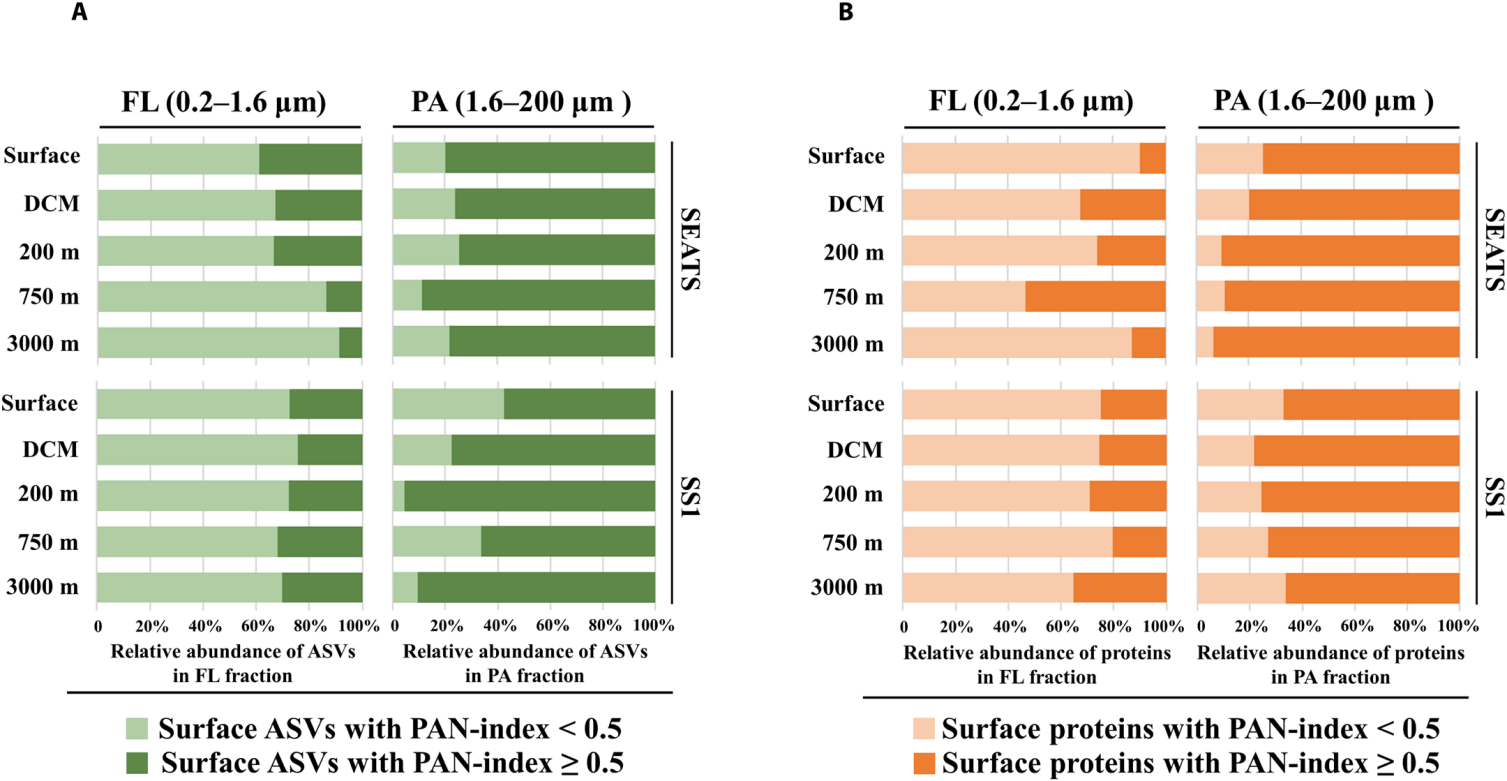
Contributions of surface ASVs and proteins enriched in large (PANindex ≥0.5 or small (PAN-index <0.5) particles to the community in a fraction from a depth. Vertical distribution of surface ASVs (**A**) and proteins (**B**) with different PAN-index values in both the FL and PA fractions along the water column at stations SEATS and SS1. Values on the horizontal axis indicate the abundance of surface ASVs or proteins accounting for a percentage of total abundance of all surface ASVs or proteins detected in a size fraction at that depth.

In this study, a particle association niche index (PAN-index) was used to indicate bacterial lifestyles and to define the size fraction preference of each ASV or protein. A PAN-index ≥0.5 indicates a higher abundance of an ASV or a protein in the PA fraction and a close association with large particles (>1.6 μm), while a PAN-index <0.5 implies enrichment of an ASV or a protein in the FL fraction, and thus, a preference for small particles (<1.6 μm) mainly including FL bacteria. Accordingly, the surface ASVs and proteins were divided into two parts and their distribution of both the FL and PA fractions throughout the water column were compared (Fig. 2). In the surface PA fraction, ASVs and proteins with a PAN-index ≥0.5 dominated, whereas in the FL fraction, ASVs and proteins with a PAN-index of <0.5 dominated. However, the surface ASVs and proteins associated with large particles (PAN-index ≥0.5) contributed 81 and 80.8%, respectively, to the average abundance in the PA fraction of deep waters, while they contributed only 25.2 and 29.3%, respectively, to the average abundance in the FL fraction (Fig. 2). In contrast, the contribution of surface ASVs and proteins with a PAN-index <0.5 was generally lower than the contribution of ASVs and proteins with a PAN-index ≥0.5 in the deep-water bacterial assemblages. The surface ASVs and proteins associated with small particles (PAN-index <0.5) contributed 74.8 and 70.7%, respectively, to the average abundance in the FL fraction of deep waters and 19.0 and 19.2% in the PA fraction (Fig. 2). These data indicate that surface microbes of both size fractions could reach the deep sea and particle size plays a role in the dispersal of surface microbes down to the deep sea.

### Seed proteins indicating viable and active surface microbes in the deep sea

Sinking particles are regarded as a relevant “seed bank” for deep-sea microbes as they are heavily colonized by microbes. We targeted those proteins present throughout the water column with a higher relative abundance in the mesopelagic and bathypelagic layers (200, 750, and 3000 m) than in the euphotic layers (surface and DCM), coined “seed proteins” (fig. S6). These proteins, as part of the surface proteins, indicated not only the functional vertical connectivity but also the potential viable and active surface microbes in the deep sea.

We identified 234 seed proteins at the SEATS station and 975 seed proteins at the SS1 station in the two size fractions, contributing 9 and 26% to the protein abundance in the surface waters of the SEATS and SS1 station, respectively, and up to 21 and 49% of the protein abundance in the deep layers of the SEATS and SS1 stations, respectively (fig. S6). Subcellular localization analysis indicated that the seed proteins at both stations were mainly located in the outer membrane, periplasm, inner membrane, and cytoplasm (fig. S7). The average proportions of cytoplasmic seed proteins at the SEATS and SS1 stations were 74.5 and 62.5%, respectively. Moreover, the average PAN-index values of seed proteins at the SEATS and SS1 stations were 0.70 and 0.47, respectively (fig. S8).

**Fig. 3.**
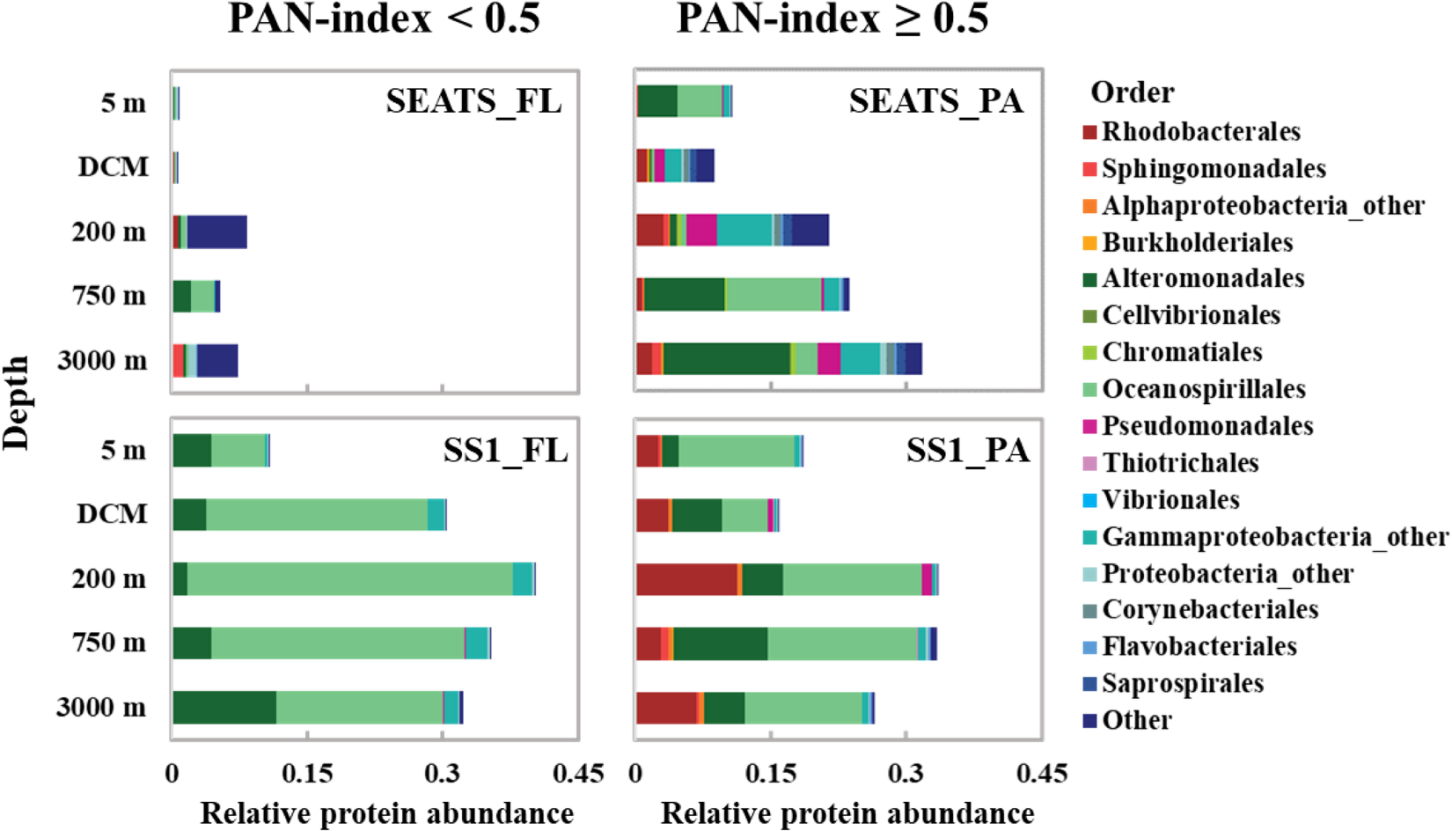
Taxonomic distribution of seed proteins in each sample at stations SEATS and SS1. The left panels (SEATS_FL and SS1_FL) show the taxonomic distribution of seed proteins with PAN-index values <0.5 in the FL fractions at stations SEATS and SS1, while the right panels (SEATS_PA and SS1_PA) show seed proteins with PAN-index values ≥0.5 in the PA fractions at the two stations.

Considering the important role of large sinking particles in mediating the functional connectivity of microbial communities, we further analyzed the taxonomic assignments of the seed proteins with a PAN-index ≥0.5 (Fig. 3). These seed proteins contributed about 14% of the total protein abundance in the surface waters, and their relative abundance increased notably with depth, contributing up to 29% in the PA fraction in the bathypelagic layer. Despite changes in the relative abundance of proteins, the taxonomic composition of seed proteins with a PAN-index ≥0.5 in the water column at the two stations was dominated mainly by Oceanospirillales, Alteromonadales, and Rhodobacterales (Fig. 3). The contribution of Oceanospirillales to seed proteins at the SEATS and SS1 station were 23.2 and 32.8%, respectively, followed by Alteromonadales (21.6 and 24.1%, respectively), and Rhodobacterales (5.2 and 25.1%, respectively). Moreover, seed proteins with a PAN-index <0.5 contributed more than 30% to the total abundance in the FL fractions in the deep sea and mainly belonged to Oceanospirillales and Alteromonadales (Fig. 3). Overall, these three taxa were not only the predominant seed protein contributors but also the most abundant functional groups in the water columns at both SEATS and SS1 stations (fig. S4). Oceanospirillales, Alteromonadales, and Rhodobacterales accounted for 11.3, 24.9, and 7.5%, respectively, of total protein abundance at the SEATS station, and 42.9, 23.0, and 14.2% at the SS1 station. In addition to these three groups, Sphingomonadales and Chromatiales also contributed to seed proteins (Fig. 3). These groups were the key microbial groups controlling the remineralization of organic matter in the water column since diverse hydrolytic enzymes were detected in these groups involved in the degradation and utilization of organic matter (fig. S9).

### Response of the surface microbes to changes experienced when transferred into the deep ocean

**Fig. 4.**
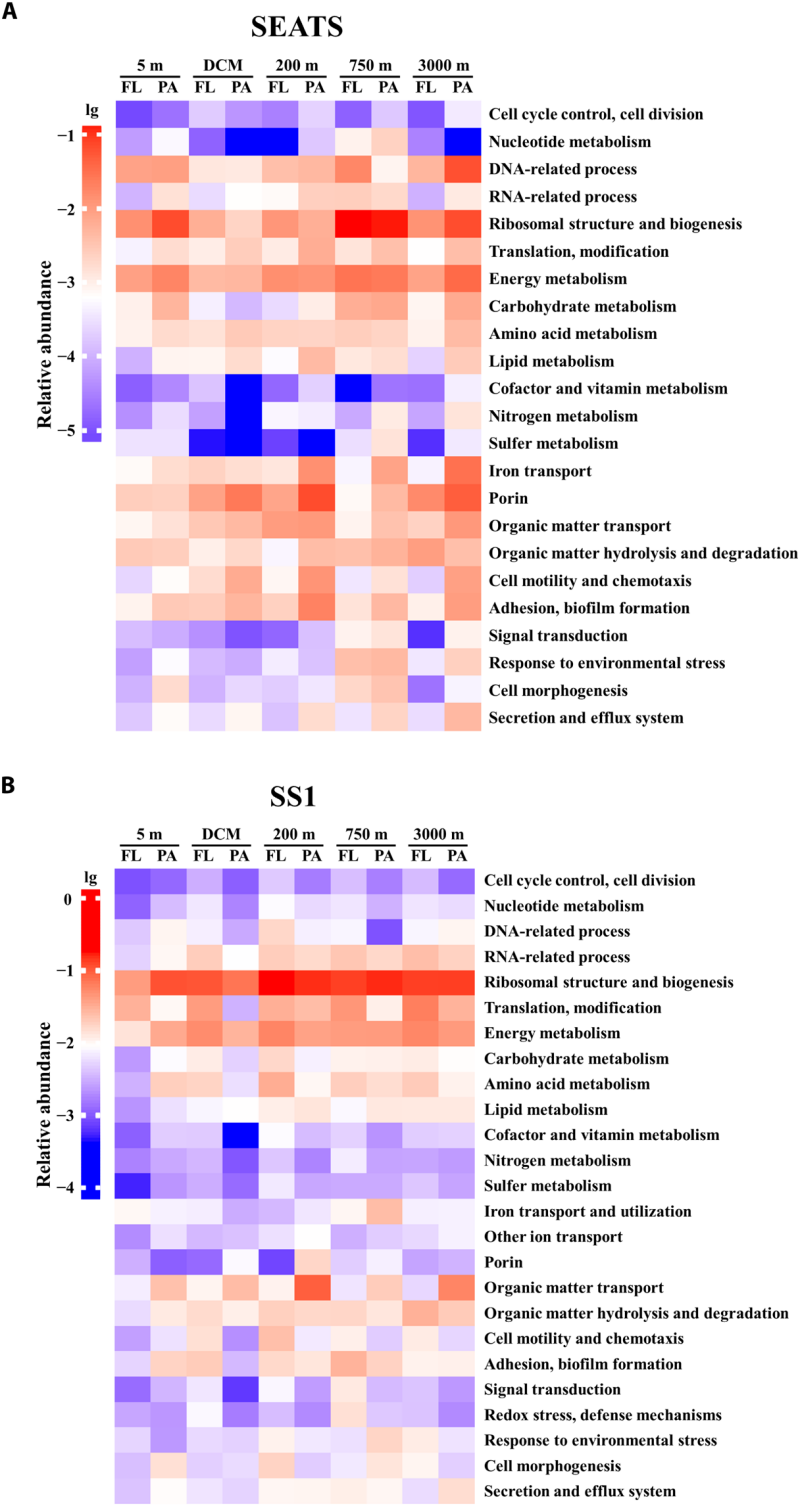
Depth-related variation of seed proteins classified by functional category. The metabolic partitioning of the seed protein expression across KEGG orthology in each sample from water column at stations SEATS (**A**) and SS1 (**B**). The relative abundance of each functional category is color coded according to their logarithmic transformation (lg10). A higher abundance is in red and lower abundance in blue.

**Fig. 5.**
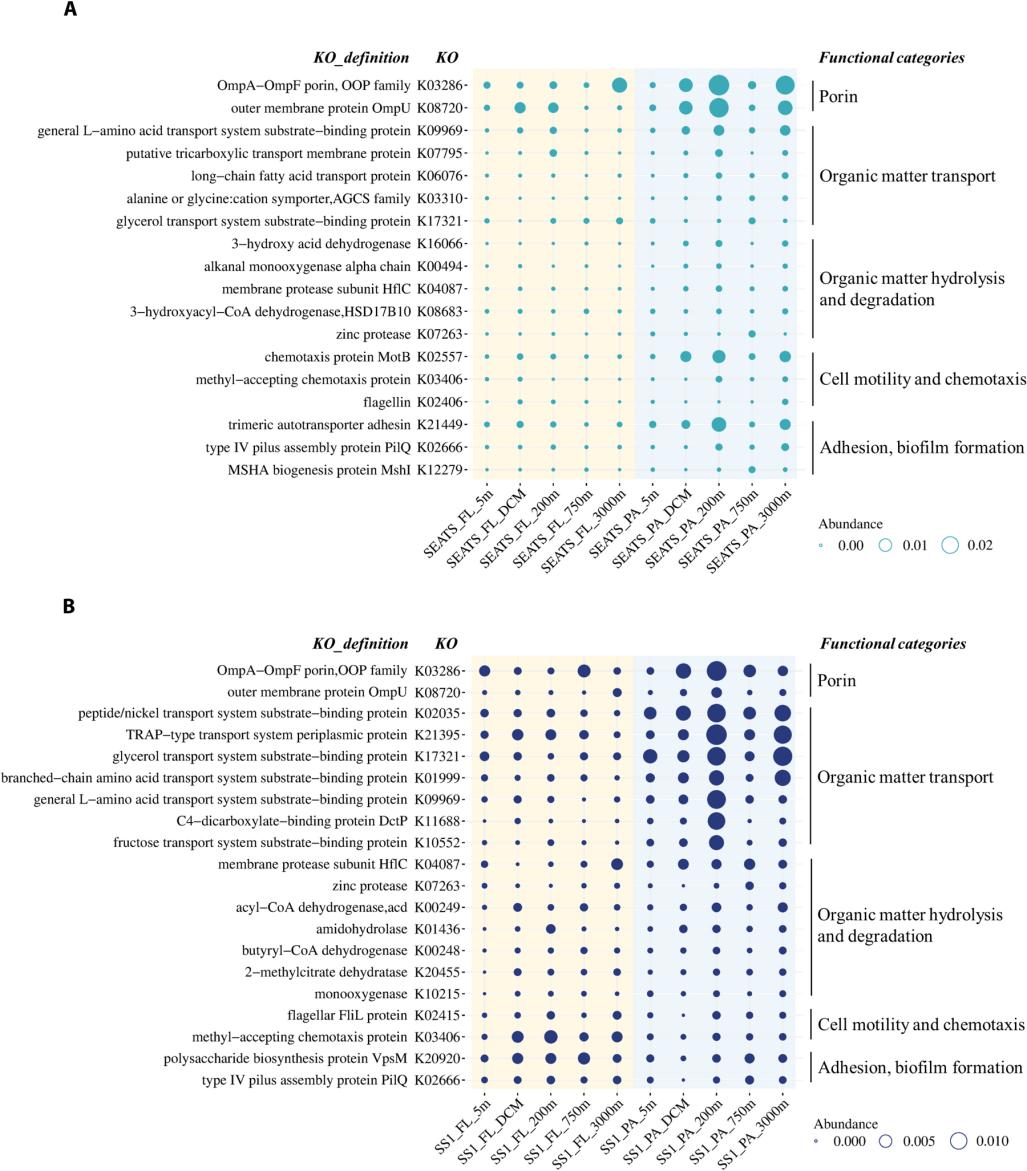
Distribution of seed proteins involved in organic matter processing. The depth-related expression pattern of representative seed proteins associated with organic matter processing at stations SEATS (**A**) and SS1 (**B**). The relative abundance of seed proteins is summed based on the same KEGG orthology and is represented by the circle size.

**Fig. 6.**
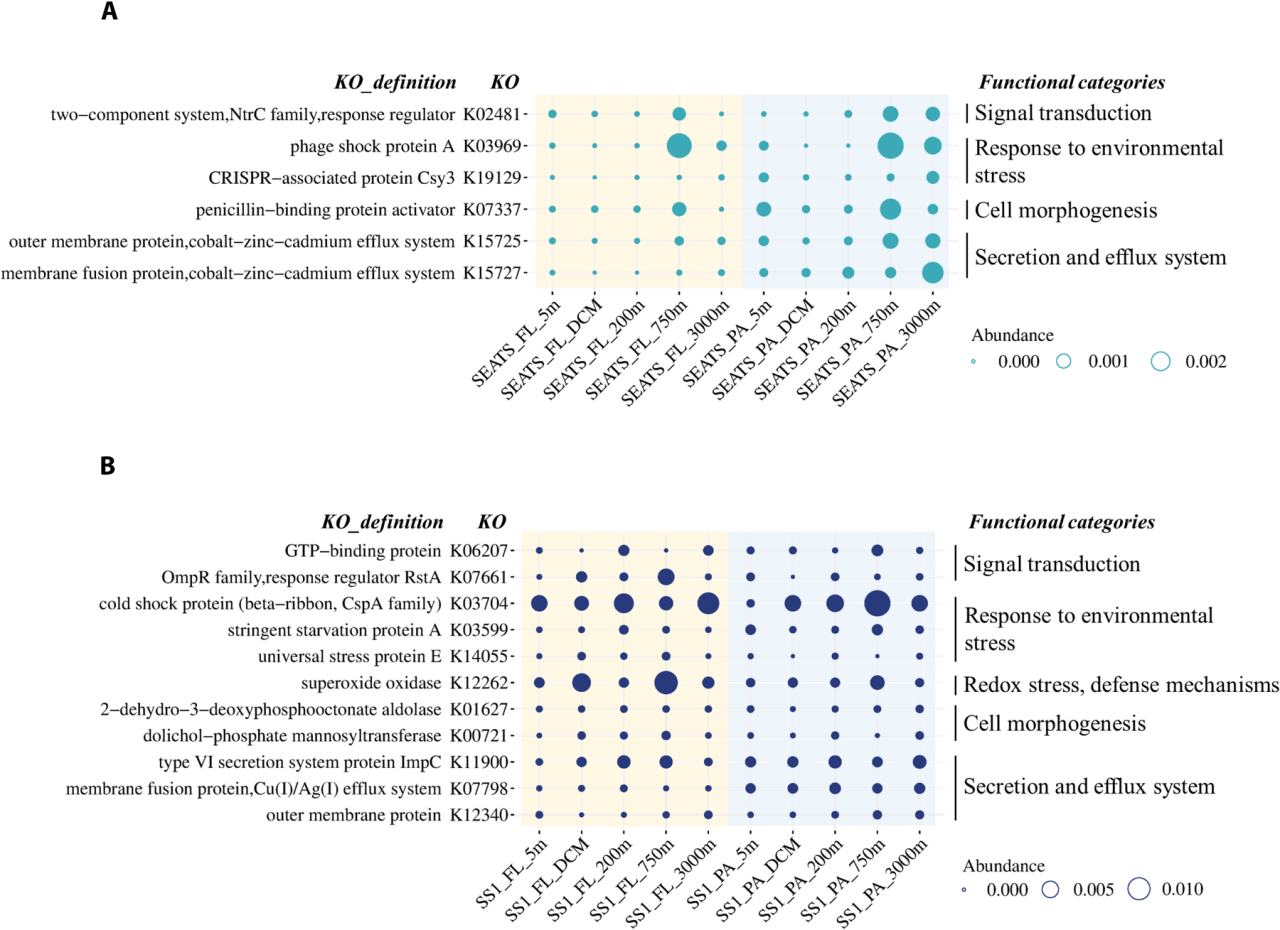
Distribution of seed proteins involved in the environmental response. The depth-related expression pattern of representative seed proteins involved in the response to environmental changes at stations SEATS (**A**) and SS1 (**B**). The relative abundance of seed proteins is summed based on the same KEGG orthology and is represented by the circle size.

We analyzed the metabolic partitioning and depth variation of the seed proteins at stations SEATS and SS1 (Figs. 4 to 6). Although there was a substantial difference in seed protein numbers at the two stations (234 versus 975), the metabolic partitioning of seed proteins was highly consistent at both stations. Depth-related variation in the seed proteins was observed in diverse functions (Fig. 4). A large proportion of seed proteins were involved in various biological processes related to basic metabolism, such as cell cycle control, cell division, nucleotide metabolism, DNA-related process, RNA-related process, ribosomal structure and biogenesis, and translation and modification. In addition, the basal cellular material and energy metabolism, such as energy metabolism, carbohydrate metabolism, amino acid metabolism, lipid metabolism, cofactor and vitamin metabolism, and essential element metabolism, including nitrogen and sulfur metabolism, and iron transport and utilization dominated the seed proteins (Fig. 4). Basic cell metabolism accounted for 65.5 and 71.9% of seed protein abundance in the mesopelagic and bathypelagic waters at stations SEATS and SS1, respectively, while the contributions of these seed proteins to total protein abundance were 10.5% in the deep waters at the SEATS station and 31.1% at the SS1 station.

A vertical pattern with a higher abundance of seed proteins in the 200- and 3000-m layers was found related to organic matter processing, such as degradation, transformation, and utilization of organic matter (Fig. 5). These seed proteins contributed to the total protein abundance from 1.8 and 4.8% in the sunlit surface waters to 4.0 and 8.1% in the deep layers at stations SEATS and SS1, respectively, accounting for 31.8 and 18.8% of seed protein abundances in the deep layers. Several seed proteins were identified as porins located in the outer membrane of bacterial cells responsible for material exchange with the surrounding environment, mainly including the OmpA-OmpF family and OmpU (Fig. 5), which might allow bacteria to transport macromolecular organic matter into the cell. In addition to porins, the highly abundant (18.3%) seed proteins were classified as transporters for a wide range of organic substrates, such as branched-chain amino acids, l-amino acids, peptides, carbohydrates such as glycerol and starch, fatty acids, and carboxylates (Fig. 5 and table S2). Moreover, seed proteins closely related to organic matter hydrolysis and degradation were also abundant (15.4%), including hydrolytic enzymes such as diverse proteases and carbohydrate hydrolases, as well as various monooxygenases, dioxygenases, and dehydrogenases (table S2). In addition, seed proteins involved in cell motility including flagellar synthesis and assembly related proteins, chemotaxis such as methyl-accepting chemotaxis proteins and chemotaxis protein MotB, adhesion and biofilm formation including pili synthesis and assembly, and biosynthesis and secretion of exopolysaccharides and adhesins were also detected (Fig. 5).

A substantial proportion of seed proteins were involved in the response to environmental changes, mainly including signal transduction, environmental stress response, cell morphogenesis, and secretion and efflux systems (Fig. 6). These seed proteins presented an increasing expression pattern in the deeper layers (750 and 3000 m), contributing 2.7 and 9.3% of seed protein abundance in the deep layers of stations SEATS and SS1, respectively. The two-component system is the most important signal transduction system in bacteria, and the seed proteins involved in signal transduction mainly belonged to the two-component system (Fig. 6). Moreover, seed proteins associated with different environmental stress responses were also identified (Fig. 6 and table S2), such as cold-shock proteins, starvation-induced proteins (e.g., stringent starvation protein A and starvation-inducible DNA binding proteins), and stress-induced proteins (e.g., the trk system potassium uptake protein, universal stress protein, CRISPR-associated protein, and phage-shock protein). In addition, seed proteins involved in redox stress and defense mechanisms exhibited strong vertical connectivity in the SS1 water column, including superoxide oxidase, superoxide dismutase, thiol peroxidase, and glutathione *S*-transferase (Fig. 6 and table S2). Seed proteins involved in cell morphogenesis, such as peptidoglycan-associated lipoprotein, penicillin-binding protein, and rod shape–determining protein were also identified (Fig. 6 and table S2). Furthermore, seed proteins involved in the secretion- and efflux-related processes (Fig. 6), such as the secretion system proteins and efflux system proteins, were detected.

## DISCUSSION

Previous studies indicate an intensive vertical connectivity of microbial community structure from the surface layers to the deep sea (*9*, *12*, *23*, *24*). Surface microbes colonizing sinking particles and being transferred to the deep sea likely determine to a considerable extent the functioning of deep-sea communities (*9*). Our results demonstrated a high functional vertical connectivity between surface and bathypelagic microbial communities in the oligotrophic South China Sea in addition to a compositional vertical connectivity. However, the vertical connectivity of microbial communities exhibited local specificity. The SS1 station exhibited a much stronger compositional and functional vertical connectivity than the SEATS station. Physicochemical parameters at the same sampling depth were comparable between the two stations (table S1). However, they still had apparent differences in hydrography (fig. S1). The SEATS station in the northern South China Sea exhibited a more dynamic and complex hydrography influenced by the Kuroshio intrusion, summer currents, and eddies. In contrast, the SS1 station in the central South China Sea was in a more stable state with less influence by water currents and eddies. Moreover, the SEATS station had the shallower depth of the surface mixed layer, nutricline, and DCM, and higher carbon export and Chl *a* concentration of DCM than the SS1 station (*25*). Thus, the different hydrographic conditions between the two stations might influence the vertical connectivity of microbial communities in the water column, and relatively stable hydrological conditions are more conducive to the vertical connectivity of microbes.

The PAN-index has been used to indicate the microbial lifestyle in a continuous niche space (*26*). In this study, surface microbes reaching the deeper layers were mainly composed of PA bacteria (PAN-index ≥0.5) in the PA fractions. This together with the results of ASVs and proteins in both surface and deep sea indicates that the particles present on the PA fraction determined to a large extent the composition and functioning of deep-sea microbial communities. This dispersal mostly influenced, but was not limited to, the bathypelagic PA communities. Similar to previous studies (*9*, *12*, *23*, *24*, *27*–*29*), PA bacteria contributed to the FL community assemblages. In addition, the small particles less than 1.6 μm (mostly composed of FL microbes) also had dispersal effects on the microbial communities of the FL fraction in the bathypelagic layer (Fig. 2). In this study, we collected samples using the Niskin bottles, which is a bulk seawater sampling method mainly collecting nonsinking particles (suspended particles and FL microbes), as well as very few sinking particles (*27*, *30*). Typically, suspended particles sink much slower than large sinking particles but these particles are still a potentially important source of the deep-sea microbial communities over a long period of sinking. Moreover, bacteria in the surface FL fraction at the surface ocean might become entrapped into large sinking particles and detach from these particles to the FL fraction when reaching the deep waters. Although the transport mechanisms might vary, our data suggest that nonsinking particles originating from the surface waters also contributed to the deep-sea microbial community. The concentration of these particles is about one to two orders of magnitude higher than that of sinking particles collected by sediment traps (*31*), and microbial metabolic activity in the dark ocean is closely tied to the suspended particles of the organic particle pool (*32*). However, thus far, the role of these particles in the biological pump has been largely ignored and their origin remains unclear. One possibility is that they are simply remnants of fragmented fast-sinking particles, such as the fragmentation of marine snow in the surface ocean by biological process and/or abiotic physical shear (*33*). Together, our results imply that sinking particles act as an important dispersal vector of viable microbes from the surface to the bathypelagic zone, ultimately mediating the functioning of deep-sea microbes.

The vertical distribution of surface ASVs and proteins indicates that surface microbes contributed the majority of the deep-sea microbial community at both stations (Fig. 1). A similar conclusion has also been reached in a study covering several oceanic provinces (*9*). Metatranscriptomic or metaproteomic studies have revealed the presence of active microbial communities in the deep sea, including allochthonous microbes from the surface ocean (*8*, *11*, *18*, *22*, *34*, *35*). The detection of seed proteins in the present study suggests that many surface microbes, such as Oceanospirillales, Alteromonadales, and Rhodobacterales, were still active in the deep sea (Fig. 3). Consistently, abundant and active prokaryotes such as Alteromonadales and Oceanospirillales are also observed in metatranscriptomes of sinking particles captured in sediment traps deployed at 4000 m depth (*11*). Our subcellular localization analysis indicated that the seed proteins were mainly located in the cytoplasm at both stations (fig. S7), implying the presence of intact cellular structures of microbes originating from surface waters in the deep layers. These cytoplasmic proteins are easily degraded without membrane protection and their abundance will decrease during sinking. Moreover, intracellular proteins involved in cellular metabolism are a proxy for the microbial response to environmental changes (*36*). Many seed proteins were identified as intracellular proteins involved in various biological processes related to basic metabolism (Fig. 4). This suggests that the microbes transported from surface waters to the deep sea maintain a highly stable basal metabolism associated with cell growth, biomolecule synthesis, and energy metabolism, allowing them to survive when facing environmental changes during sinking through the water column and retaining their metabolic functions even in the bathypelagic ocean.

The bioavailability of organic substrates is critical for microbial survival in the deep oceanic water column due to the decrease in concentration and nutritive quality of organic matter with depth (*37*–*40*). Thus, surface microbes are faced with increasing challenges of nutrient acquisition as they descend to the deep ocean. We found a depth-related expression of a considerable part of seed proteins associated with degradation, transformation, and utilization of organic matter, such as porin, organic matter transport, organic matter hydrolysis, and degradation (Figs. 4 and 5). These proteins play important roles in the processing of organic compounds in the deep sea (*41*, *42*). Similar to previous studies (*8*, *35*), the expression level of transporters changed with depth, suggesting the presence of depth-related patterns in the concentration of bioavailable substrates. A part of marine microbes is motile and hence, able to approach organic “hot spots” through expressing flagellin and chemotaxis proteins. This allows them to attach to particles using pilin and to form biofilms by secreting exopolysaccharides and adhesins, ultimately allowing for a better utilization of particles as carbon and energy source (*43*–*45*). In our study, seed proteins involved in cell motility and chemotaxis, adhesion, and biofilm formation were more abundant at the depths where transporters were also abundant (Figs. 4 and 5). These results suggest that the microbes enhanced the utilization efficiency of organic substrates during their dispersal from surface waters to the deep sea, as indicated by the expression of transporters and enzymes tightly related to degradation, transformation, and transport of organic matter. In addition, these surface water–derived microbes enhanced their motility, chemotaxis, and adhesion to converge on organic hot spots and thus ensuring the effective utilization of deep-sea POM. These results also imply that the surface microbes transferred to the deep-sea realm prefer a PA lifestyle. In contrast to the low concentration and recalcitrant feature of the dissolved organic matter pool in the deep ocean, the deep-sea POM pool has a higher nutritive value and thus is more suitable for meeting the carbon and energy demands of the microbes in the ocean’s interior (*22*, *26*).

During the descent through the water column from the surface to the deep sea, surface microbes are faced with various survival challenges caused by marked environmental changes along the water column, such as light, temperature, dissolved oxygen, and hydrostatic pressure (*3*, *14*, *15*, *18*). Studies have shown that marine bacteria have evolved diverse mechanisms in response to environmental changes, via regulating expressions of proteins associated with signal transduction (*46*, *47*), anticold (*20*), antistarvation (*48*), redox stress and defense (*18*, *19*, *49*–*51*), cell morphology maintenance (*52*–*54*), and the secretion- and efflux-related process (*44*, *55*, *56*). These mechanisms protect them from environmental damage and allow them to survive under less-than-optimal living conditions. In our study, we found that abundances of many seed proteins involved in stress-response processes exhibited depth-related patterns with higher expression in deep waters, especially in the 750-m layer which was characterized by the lowest dissolved oxygen concentrations (2.75 and 2.69 mg/liter at stations SEATS and SS1, respectively). Proteins, such as NtrC family response regulator, guanosine triphosphate–binding protein, cold-shock protein, phage-shock protein, and superoxide oxidase presented the highest relative abundance at this depth, indicating that dissolved oxygen is a critical stressor for surface microbes but these microorganisms can actively respond to low dissolved oxygen via increasing expression of proteins associated with stress response. Notably, approximately 80% of deep-sea microbes are insensitive to hydrostatic pressure changes (*18*) although rapid changes in hydrostatic pressure lead to an inhibition of the metabolic activity (*57*). In our study, we did not detect proteins associated with hydrostatic pressure response. Therefore, insensitivity to hydrostatic pressures might be an essential survival strategy of surface microbes, which guarantees them to survive at different hydrostatic pressures during the sinking from the surface to the deep sea. Collectively, the diverse responses of the surface-derived microbes to specific environmental stresses during the descent in the water column are not only mediated through regulating the signal transduction system (e.g., two-component system) but also by initiating specific defense mechanisms in response to various environmental stressors, e.g., temperature, starvation, and dissolved oxygen. Thus, surface-derived microbes can actively adjust their metabolic behaviors in response to the marked environmental changes during their sinking from surface waters to the deep sea and ultimately survive in the deep sea.

In summary, we demonstrate both the functional and compositional vertical connectivity of microbial communities from surface to deep waters. In particular, our findings of seed proteins highlight that the surface water–derived microbes are capable of actively adjusting their metabolic strategies in response to the environmental changes during the transfer from the surface to the deep sea by maintaining a highly stable basal metabolism and enhancing the transport and utilization efficiency of organic matter in the deep sea. Moreover, these microbes activate depth-related defense responses to various environmental stressors to allow their survival in deep waters and likely contribute to the functioning of deep ocean microbial communities. However, efforts should be made to investigate whether these findings regarding functional vertical connectivity of microbial communities reported in the South China Sea are also valid in other oceanic regions with different hydrographic conditions. Moreover, comparative studies of the diversity and metabolic characteristics of microbes from sediment traps and suspended particles will help to better constrain the extent of the functional vertical connectivity of microbial communities. In addition, the contribution of surface microbes to the functioning of deep-sea microbial communities needs to be quantified using protein-based stable-isotope probing and/or targeted quantitative metaproteomic approach combined with biogeochemical process measurements. Last, the characterization of species/strains from Oceanospirillales, Alteromonadales, and Rhodobacterales isolated at different water depths is critical for verifying our findings. The results of these studies will revolutionize our understanding of the ecological and biogeochemical roles of surface water–derived microbes in the deep ocean.

## MATERIALS AND METHODS

### Study area and sampling

The survey was conducted during the CHOICE-II KK1702 cruise in the South China Sea in June 2017. Microbial samples were collected at two stations, SEATS (18°N, 116°E) and SS1 (14°N, 116°E), located in the north and center of the South China Sea basin, respectively (fig. S1). The sampling depths included 5, DCM (75 m at the SEATS station and 100 m at the SS1 station), 200, 750, and 3000 m. Seawater was collected using Niskin bottles deployed on a rosette containing a conductivity-temperature-depth profiler (Sea Bird Electronics, Washington, USA) equipped with a dissolved oxygen sensor, fluorometer, and transmissometer. Phosphate, silicate, nitrate, and nitrite concentrations were determined using a Skalar San++ continuous flow analyzer (*58*). A size-fractionated sampling strategy was adopted to collect both a large-size fraction (1.6 to 200 μm) and small-size fraction (0.2 to 1.6 μm) of the microbial community. This strategy involved prefiltering the seawater through a 200-μm filter to remove large zooplankton. Microbial biomass for metaproteomics and 16*S* rRNA analyses was then collected via sequential in-line filtration of seawater through a 1.6-μm pore size prefilter (GF/A, 47 mm diameter, Whatman) and then a 0.2-μm pore size filter (PES, 47 mm diameter, Whatman) using a peristaltic pump. The small-size fraction microbes ranging from 0.2 to 1.6 μm were expected to predominantly contain FL prokaryotes, while the large-size fraction microbes ranging from 1.6 to 200 μm mainly retained PA prokaryotes (*59*). Sampling for metagenomics was also performed during the cruise and the size fraction collected ranged from 0.2 to 200 μm. The collected samples were immediately frozen in liquid nitrogen and stored at −80°C until DNA and protein extraction.

### Total DNA extraction, 16*S* rRNA analysis, and metagenome sequencing

Total DNA was extracted following a modified cetyltrimethylammonium bromide (CTAB) method (*60*, *61*). In brief, filters immersed in CTAB buffer were incubated at 60°C for 1 hour. Then, the extracted DNA was purified and RNase digested to remove contaminating RNA from DNA. After the steps of precipitation and washing, the DNA was dissolved in 100 μl of Milli-Q water and stored at −80°C until sequencing.

The V3-V4 region of 16*S* rRNA gene was amplified using the bacterial universal primers 338F (5′-ACTCCTACGGGAGGCAGCA-3′) and 806R (5′-GGACTACHVGGGTWTCTAAT-3′) (*62*, *63*). The polymerase chain reaction was performed in 20-μl reaction volumes and TransStart Fastpfu DNA Polymerase (TransGen AP221-02) was added. Amplicons were extracted using 2% agarose gels and purified with the AxyPrep DNA Gel Extraction Kit (Axygen, CA, USA) according to the manufacturer’s instructions. The quantification was performed on the QuantiFluor-ST System (Promega, USA). The purified amplicons were paired-end sequenced on an Illumina MiSeq platform following the standard protocols. Raw sequence reads obtained in this study were deposited in the National Center for Biotechnology Information (NCBI) database under the BioProject accession number PRJNA989431. After demultiplexing, raw reads were quality-filtered using Fastp (version 0.19.6). Sequences were assembled based on their overlap sequence using Flash (version 1.2.7). After sequence denoising using the DADA2 R package, ASVs were obtained and used for the following bioinformatics analysis. The observed richness (alpha diversity) was calculated using the online tool of Majorbio Cloud Platform (https://cloud.majorbio.com/page/tools/) (*64*).

Metagenomic library construction was conducted according to the manufacturer’s protocol. Shotgun sequencing was performed on an Illumina Hiseq X-Ten platform (Illumina, CA, USA). Raw reads were filtered using SOAPnuke (version 1.5.6) to remove adaptor contamination and low-quality and duplicate reads. Clean reads were assembled using the IDBA-UD program (version 1.1.2). Open reading frames (ORFs) in contigs of each sample were predicted by MetaGeneMark (version 3.38). The nonredundant gene set was generated using CD-HIT (version 4.6) based on ORFs and then translated into protein reference database for the subsequent metaproteomics analysis. Raw data from this study have been deposited in the China National GeneBank (CNGB) DataBase (https://db.cngb.org) via the CNGB Nucleotide Sequence Archive under accession number CNP0001000.

### Protein extraction, separation, and liquid chromatography–tandem mass spectrometry analysis

Protein extraction was performed as previous described (*61*, *65*). In brief, filter membranes with 20 ml of TRIzol reagent were homogenized. Samples were subsequently centrifuged. Then, the supernatant was transferred and mixed with 4 ml of chloroform and then held at room temperature for 5 min. After centrifugation, the reddish bottom layer was resuspended in 6 ml of ethanol, vortexed, and centrifuged. The resulting supernatant was mixed with isopropanol and stored at −20°C overnight. After centrifugation and washing, the obtained pellet was air-dried and dissolved in a rehydration buffer. Protein concentration was determined using a 2D Quant kit (GE Healthcare, USA). Protein separation and liquid chromatography–tandem mass spectrometry (MS) analysis were conducted following a previously reported protocol (*61*).

### Protein identification and bioinformatics analysis

Tandem mass spectra were searched against a protein database predicted from metagenomes of the South China Sea obtained from the same cruise. Protein identification and quantification from the MS raw data were performed with the MetaPro-IQ approach as previously described (*66*). In brief, the protein database was generated with a two-step iterative search strategy using X!Tandem (2017.2.1 version), and then protein identification and label-free quantification were conducted using MaxQuant (1.6.1.0 version) (*67*). A stringent cutoff of the false discovery rate at <1% was used for identification at both peptide-spectra match and protein level. Proteins matching at least two unique peptides were considered as high-confidence identification and selected for further bioinformatic analysis. MaxQuant results and MS raw files were deposited in the ProteomeXchange Consortium (http://proteomecentral.proteomexchange.org) via the PRoteomics IDEntifications (PRIDE) partner repository with dataset identifiers PXD018513 and PXD026838. High-confidence proteins were searched against the NCBI nonredundant protein sequence database (v20170924) using the basic local alignment search tool (BLAST), and the top 10 hits were recorded. BLAST search results were loaded into MEGAN, and taxonomic assignment was performed using the lowest common ancestor algorithm (bit score > 80) (*68*). All the quantified protein sequences were aligned against the Kyoto Encyclopedia of Genes and Genomes (KEGG) database (version 81) using BLAST (*69*) with default parameters (*e* value cut-off at 1 × 10^−5^). The subcellular localization of identified proteins was predicted using the Protein Subcellular Localization Prediction Tool (*70*). The nonmetric multidimensional scaling ordinations representing spatially the Bray-Curtis distances between the microbial communities and between their protein expressions were analyzed using the online tools on the Majorbio Cloud Platform (https://cloud.majorbio.com/page/tools/) (*64*). To measure the position of surface ASV and protein in a continuous niche space ranging from a completely FL lifestyle to a completely PA lifestyle, the PAN-index for surface ASV or protein was calculated by an abundance-weighted mean according to a previous study (*26*). In brief, the abundance of a given ASV or protein in each sample and in each size fraction was recorded. A value of 0 was assigned to FL samples, while a value of 1 was assigned to PA samples. Then, the abundance-weighted mean of each ASV or protein was calculated as its PAN-index. Thus, a PAN-index <0.5 indicates a preference for a FL lifestyle, while a PAN-index of ≥0.5 indicates a preference for a particle-associated lifestyle.
