# Supplementary material for: Functional vertical connectivity of microbial communities in the ocean
Source: Sci Adv. 2024 May 23;10(21):eadj8184. doi: 10.1126/sciadv.adj8184 (PMC11114224; doi:10.1126/sciadv.adj8184)
Supplement: Supplementary file 1 — Figs. S1 to S9 Table S1 Legend for table S2 [file sciadv.adj8184_sm.pdf]

Science Advances
AAAS

Supplementary Materials for

Functional vertical connectivity of microbial communities in the ocean

Shi Chen *et al.*

Corresponding author: Gerhard J. Herndl, gerhard.herndl@univie.ac.at; Da-Zhi Wang, dzwang@xmu.edu.cn

Sci. Adv. **10**, eadj8184 (2024)
DOI: 10.1126/sciadv.adj8184

The PDF file includes:

Figs. S1 to S9
Table S1
Legend for table S2

Other Supplementary Material for this manuscript includes the following:

Table S2

Supplementary Materials

Fig. S1

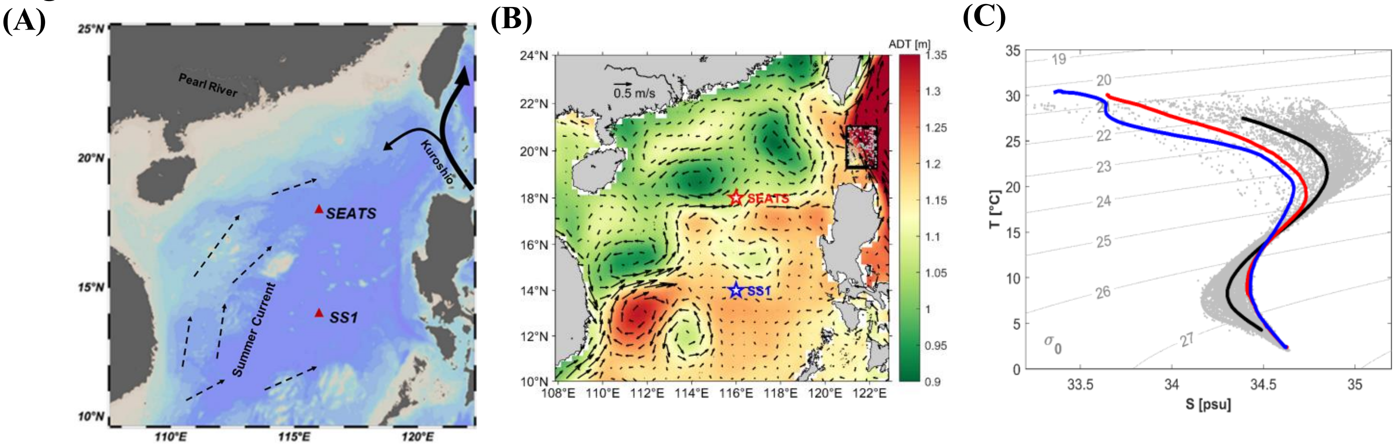

Fig. S1. Hydrography of stations SEATS and SS1 during the survey of June 2017. (A) The SEATS station is located in the northern South China Sea and the SS1 station is in the central South China Sea. During the survey, the hydrography of the SEATS station was more dynamic and complex influenced by Kuroshio intrusion and summer currents, while the SS1 station was in a more stable state uninfluenced by water mass and currents. **(B)** Absolute dynamic topography (ADT; colored) and its associated geostrophic velocities (vectors) during the survey of June 2017. The SEATS station was located at the edge of a cyclonic eddy while the SS1 station was situated in a relatively calm area uninfluenced by the eddy. The gray dots in the black box denoted the positions of the Argo floats probing the Kuroshio current in the Luzon Strait. **(C)** Temperature-salinity diagram of water properties in stations SEATS (red line) and SS1 (blue line). The water properties of the SEATS station were closer to the characteristics of the Kuroshio current. Gray dots, representing the Kuroshio current, were based on Argo measurements with the mean values denoted by the thick black line. The red and blue lines were based on *in situ* CTD measurements collected in stations SEATS and SS1, respectively. The satellite altimeter data (including the ADT and its geostrophic velocities) during the survey period were obtained from the Copernicus Marine Environment Monitoring Service (CMEMS; http://marine.copernicus.eu/). The Argo profiles for the period 2004 to 2022 were obtained from Euro-Argo (https://dataselection.euro-argo.eu/).

Fig. S2

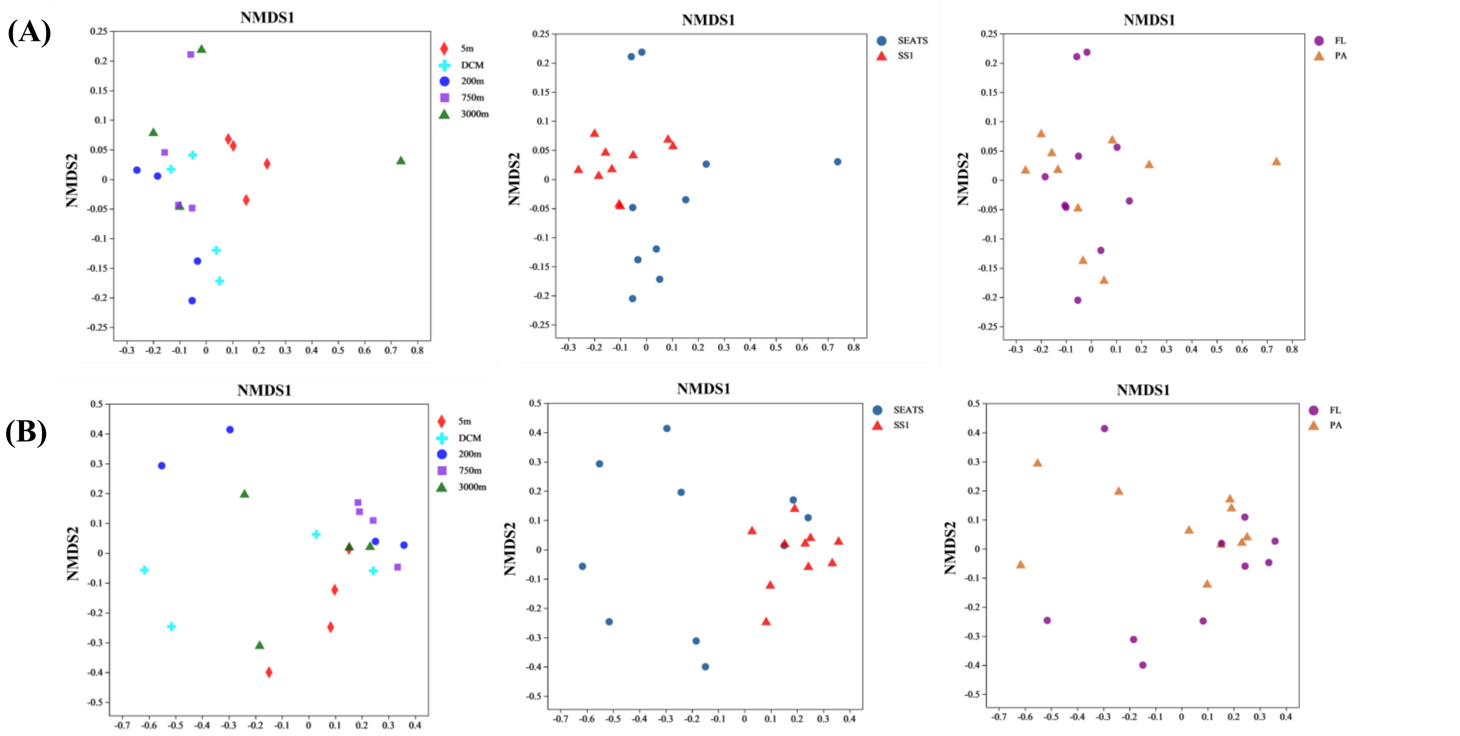

Fig. S2. Nonmetric multidimensional scaling (nMDS) ordinations represent spatially the Bray-Curtis distances between the microbial communities (A) and between the protein expressions (B). Distances are calculated using the rarefied ASVs and the normalized protein abundance, respectively. Samples are color-coded based on sampling depth (5 m, DCM, 200 m, 750 m, and 3,000 m), station (SEATS and SS1) and size fraction (FL, 0.2-1.6 µm; PA, 1.6-200 µm).

Fig. S3

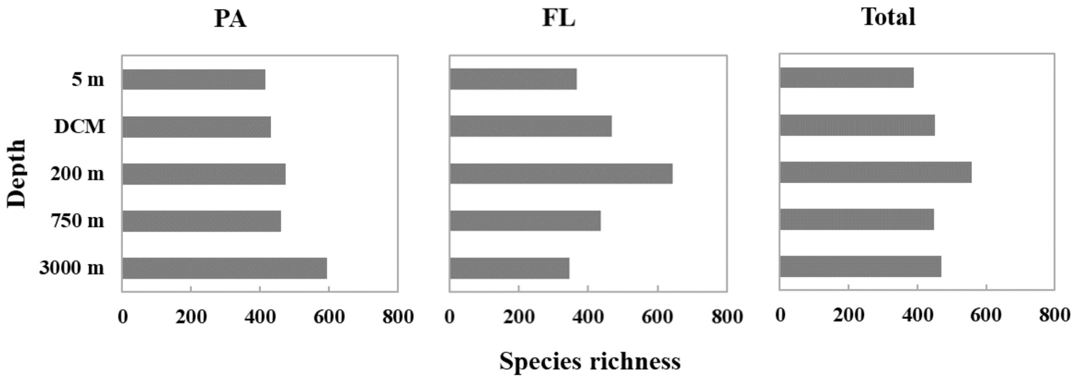

Fig. S3. The observed species richness (ASV-based) of microbial communities in free-living (FL), particle-associated (PA) and combined (Total) fractions in each depth of two stations. The combined fraction is the sum of free-living and particle-associated fractions.

Fig. S4

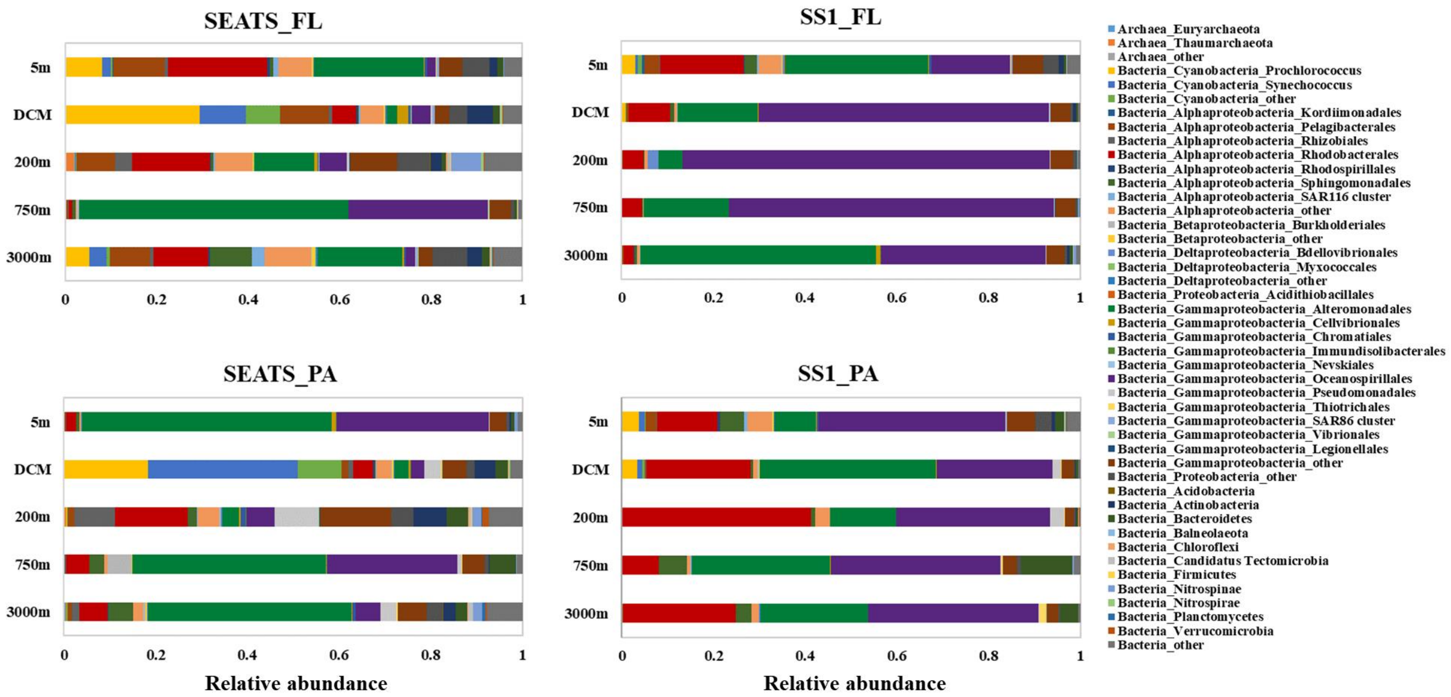

Fig. S4. Metaproteome-based microbial community structure of the FL and PA fractions at each depth (5 m, DCM, 200 m, 750 m and 3,000 m) of stations SEATS and SS1. The horizontal axis indicates the relative abundance of seed proteins classified by microbial group.

Fig. S5

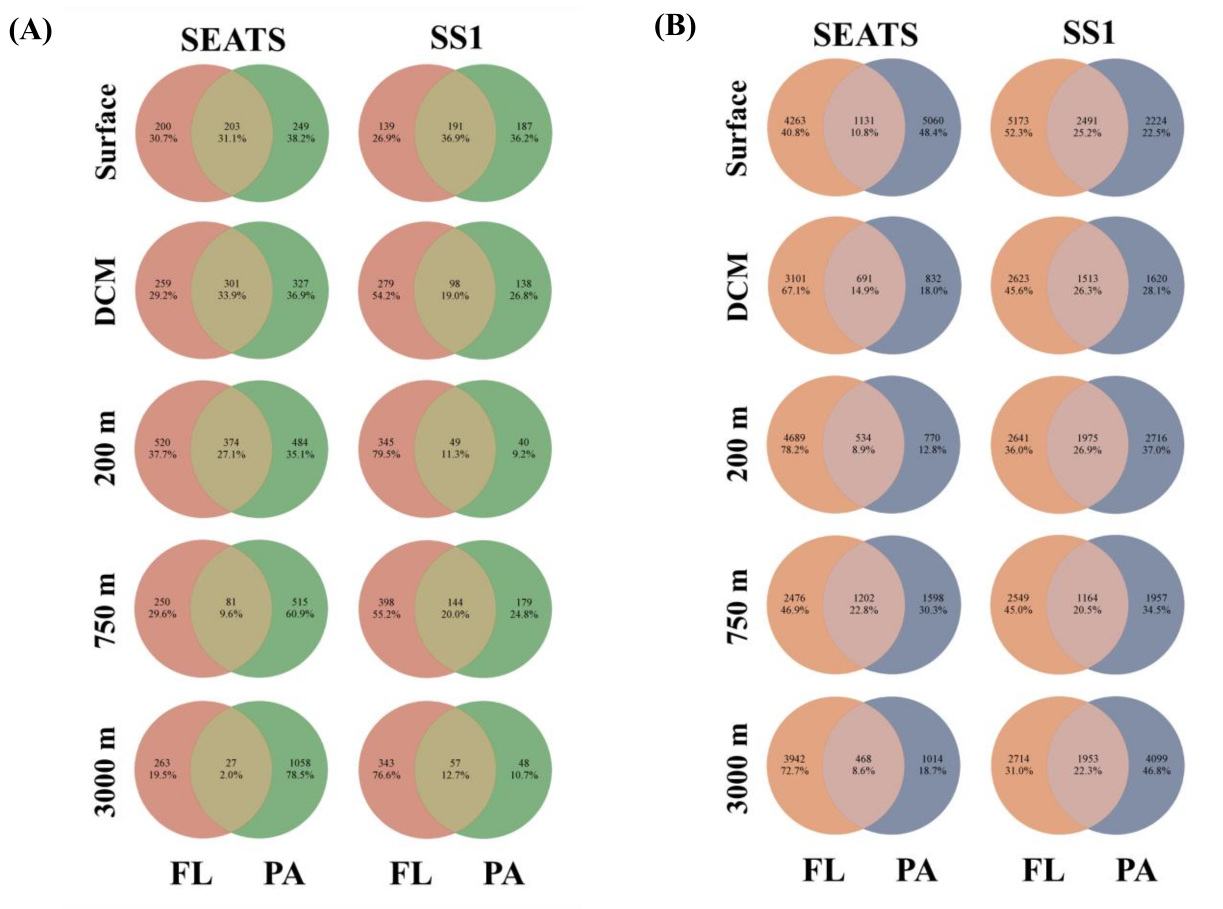

Fig. S5. Venn diagrams illustrating shared ASVs (A) and proteins (B) between the FL fraction and the PA fraction at each depth (5 m, DCM, 200 m, 750 m, and 3,000 m) of stations SEATS and SS1. Values in pie charts indicate the counts and the percentage of ASVs and proteins in each part.

Fig. S6

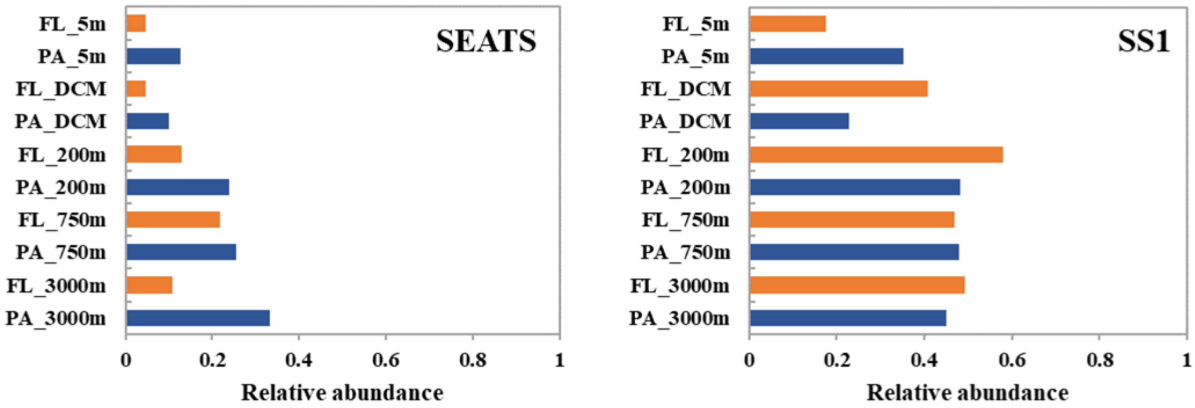

Fig. S6. Vertical distribution of seed proteins detected in FL and PA fractions at stations SEATS (left) and SS1 (right). The horizontal axis indicates the accumulative relative abundance of seed proteins in each sample.

Fig. S7

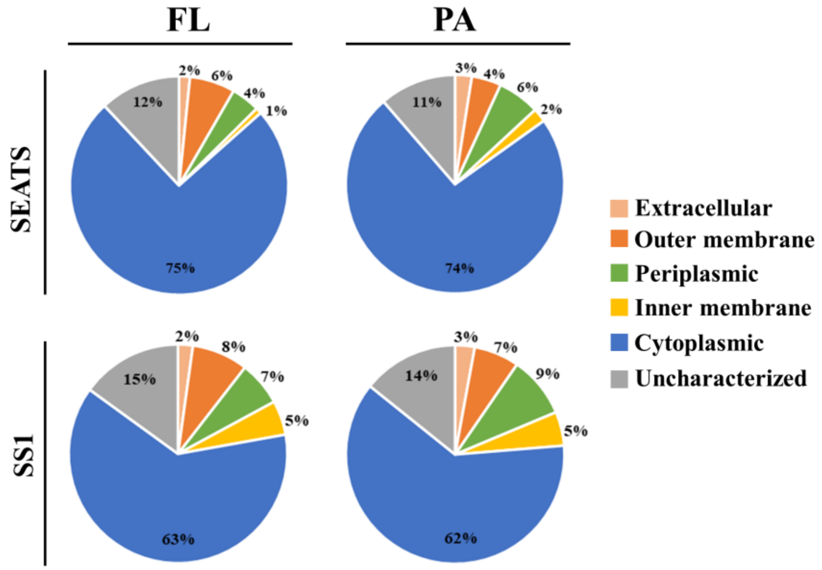

Fig. S7. Distribution of seed proteins classified by predicted subcellular localization in the FL and PA fractions at stations SEATS and SS1. The percentage of each category in each pie chart is based on the average of its relative abundances in all depths.

Fig. S8

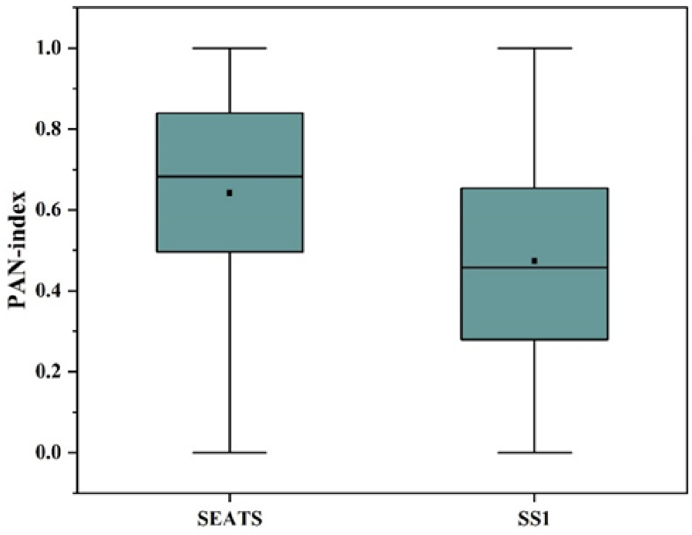

Fig. S8. The particle-association niche index (PAN-index) of seed proteins in the SEATS and SS1 water columns. The PAN-index is calculated by an abundance-weighted mean according to a previous study (26).

Fig. S9

(A)

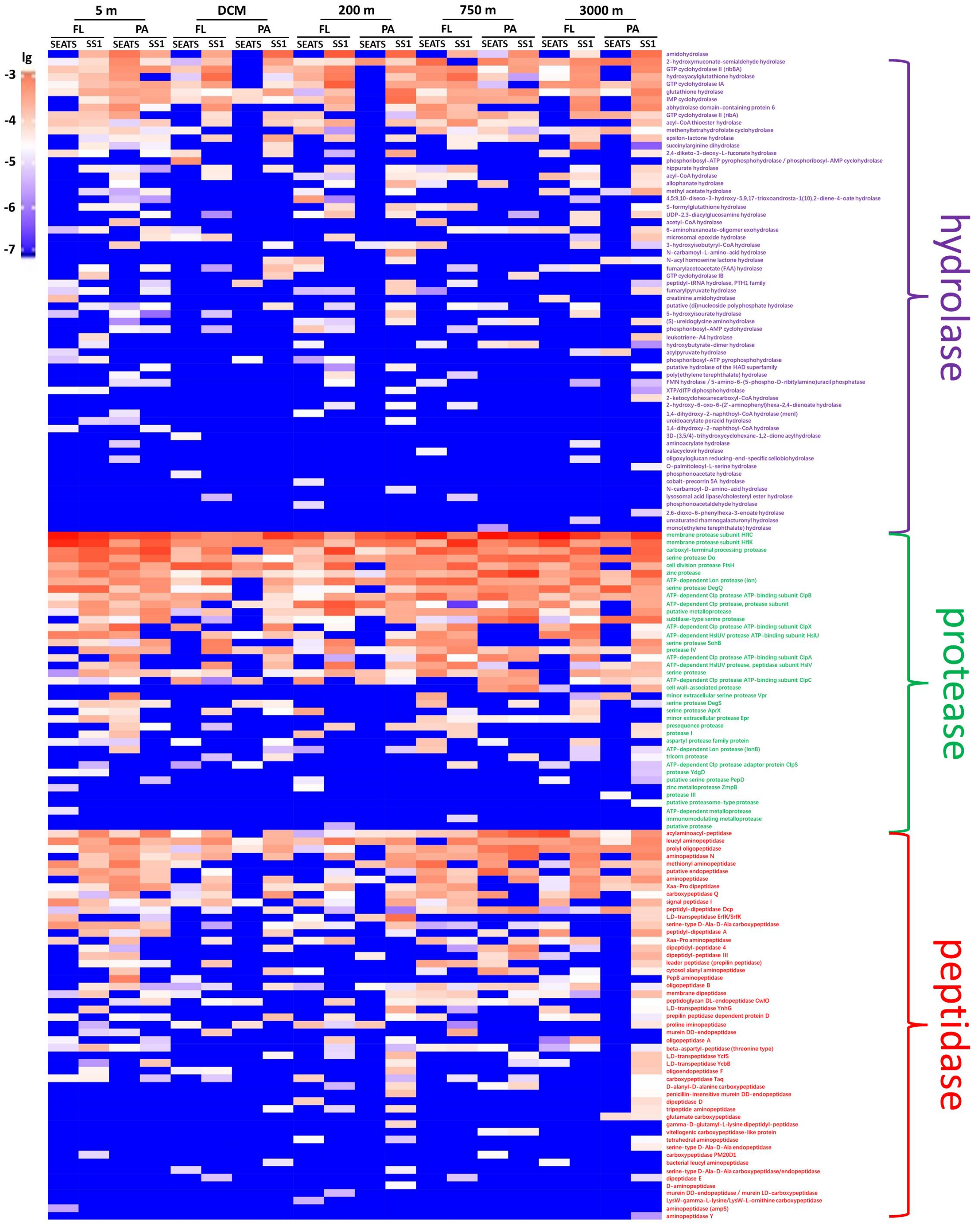
5 m
DCM
200 m
750 m
3000 m
FL
PA
SEATS
SS1
lg
-3
-4
-5
-6
-7
amidohydrolase
2-hydroxymuconate-semialdehyde hydrolase
GTP cyclohydrolase II (ribBA)
hydroxyacylglutathione hydrolase
GTP cyclohydrolase IA
glutathione hydrolase
IMP cyclohydrolase
abhydrolase domain-containing protein 6
GTP cyclohydrolase II (ribA)
acyl-CoA thioester hydrolase
methenyltetrahydrofolate cyclohydrolase
epsilon-lactone hydrolase
succinylarginine dihydrolase
2,4-diketo-3-deoxy-L-fuconate hydrolase
phosphoribosyl-ATP pyrophosphohydrolase / phosphoribosyl-AMP cyclohydrolase
hippurate hydrolase
acyl-CoA hydrolase
allophanate hydrolase
methyl acetate hydrolase
4,5:9,10-diseco-3-hydroxy-5,9,17-trioxoandrosta-1(10),2-diene-4-oate hydrolase
S-formylglutathione hydrolase
UDP-2,3-diacylglucosamine hydrolase
acetyl-CoA hydrolase
6-aminohexanoate-oligomer exohydrolase
microsomal epoxide hydrolase
3-hydroxyisobutyryl-CoA hydrolase
N-carbamoyl-L-amino-acid hydrolase
N-acyl homoserine lactone hydrolase
fumarylacetoacetate (FAA) hydrolase
GTP cyclohydrolase IB
peptidyl-tRNA hydrolase, PTH1 family
fumarylpyruvate hydrolase
creatinine amidohydrolase
putative (di)nucleoside polyphosphate hydrolase
5-hydroxyisourate hydrolase
(S)-ureidoglycine aminohydrolase
phosphoribosyl-AMP cyclohydrolase
leukotriene-A4 hydrolase
hydroxybutyrate-dimer hydrolase
acylpyruvate hydrolase
phosphoribosyl-ATP pyrophosphohydrolase
putative hydrolase of the HAD superfamily
poly(ethylene terephthalate) hydrolase
FMN hydrolase / 5-amino-6-(5-phospho-D-ribitylamino)uracil phosphatase
XTP/dITP diphosphohydrolase
2-ketocyclohexanecarboxyl-CoA hydrolase
2-hydroxy-6-oxo-6-(2'-aminophenyl)hexa-2,4-dienoate hydrolase
1,4-dihydroxy-2-naphthoyl-CoA hydrolase (menI)
ureidoacrylate peracid hydrolase
1,4-dihydroxy-2-naphthoyl-CoA hydrolase
3D-(3,5/4)-trihydroxycyclohexane-1,2-dione acylhydrolase
aminoacrylate hydrolase
valacyclovir hydrolase
oligoxyloglucan reducing-end-specific cellobiohydrolase
O-palmitoleoyl-L-serine hydrolase
phosphonoacetate hydrolase
cobalt-precorrin 5A hydrolase
N-carbamoyl-D-amino-acid hydrolase
lysosomal acid lipase/cholesteryl ester hydrolase
phosphonoacetaldehyde hydrolase
2,6-dioxo-6-phenylhexa-3-enoate hydrolase
unsaturated rhamnogalacturonyl hydrolase
mono(ethylene terephthalate) hydrolase
membrane protease subunit HflC
membrane protease subunit HflK
carboxyl-terminal processing protease
serine protease Do
cell division protease FtsH
zinc protease
ATP-dependent Lon protease (lon)
serine protease DegQ
ATP-dependent Clp protease ATP-binding subunit ClpB
ATP-dependent Clp protease, protease subunit
putative metalloprotease
subtilase-type serine protease
ATP-dependent Clp protease ATP-binding subunit ClpX
ATP-dependent HslUV protease ATP-binding subunit HslU
serine protease SohB
protease IV
ATP-dependent Clp protease ATP-binding subunit ClpA
ATP-dependent HslUV protease, peptidase subunit HslV
serine protease
ATP-dependent Clp protease ATP-binding subunit ClpC
cell wall-associated protease
minor extracellular serine protease Vpr
serine protease DegS
serine protease AprX
minor extracellular protease Epr
presequence protease
protease I
aspartyl protease family protein
ATP-dependent Lon protease (lonB)
tricorn protease
ATP-dependent Clp protease adaptor protein ClpS
protease YdgD
putative serine protease PepD
zinc metalloprotease ZmpB
protease III
putative proteasome-type protease
ATP-dependent metalloprotease
immunomodulating metalloprotease
putative protease
acylaminoacyl-peptidase
leucyl aminopeptidase
prolyl oligopeptidase
aminopeptidase N
methionyl aminopeptidase
putative endopeptidase
aminopeptidase
Xaa-Pro dipeptidase
carboxypeptidase Q
signal peptidase I
peptidyl-dipeptidase Dcp
L,D-transpeptidase ErfK/SrfK
serine-type D-Ala-D-Ala carboxypeptidase
peptidyl-dipeptidase A
Xaa-Pro aminopeptidase
dipeptidyl-peptidase 4
dipeptidyl-peptidase III
leader peptidase (prepilin peptidase)
cytosol alanyl aminopeptidase
PepB aminopeptidase
oligopeptidase B
membrane dipeptidase
peptidoglycan DL-endopeptidase CwlO
L,D-transpeptidase YnhG
prepilin peptidase dependent protein D
proline iminopeptidase
murein DD-endopeptidase
oligopeptidase A
beta-aspartyl-peptidase (threonine type)
L,D-transpeptidase YcfS
L,D-transpeptidase YcbB
oligoendopeptidase F
carboxypeptidase Taq
D-alanyl-D-alanine carboxypeptidase
penicillin-insensitive murein DD-endopeptidase
dipeptidase D
tripeptide aminopeptidase
glutamate carboxypeptidase
gamma-D-glutamyl-L-lysine dipeptidyl-peptidase
vitellogenic carboxypeptidase-like protein
tetrahedral aminopeptidase
serine-type D-Ala-D-Ala endopeptidase
carboxypeptidase PM20D1
bacterial leucyl aminopeptidase
serine-type D-Ala-D-Ala carboxypeptidase/endopeptidase
dipeptidase E
D-aminopeptidase
murein DD-endopeptidase / murein LD-carboxypeptidase
LysW-gamma-L-lysine/LysW-L-ornithine carboxypeptidase
aminopeptidase (ampS)
aminopeptidase Y
hydrolase
protease
peptidase

(B)

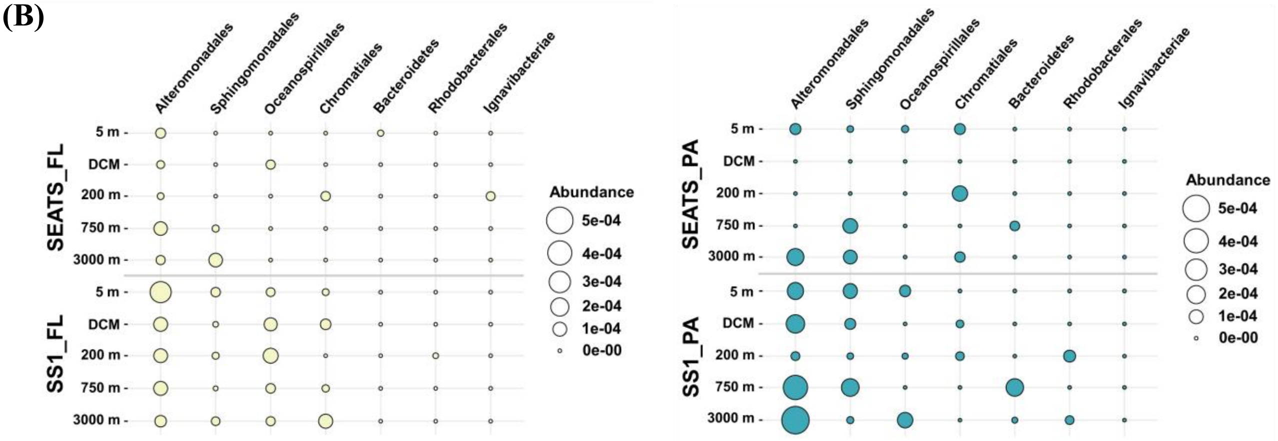

Fig. S9. Hydrolytic enzymes detected in the water columns of stations SEATS and SS1. (A) Relative abundance and categories of hydrolytic enzymes are shown for each sample with information of size fraction, depth and station. The relative abundance of each enzyme is color coded according to the base-10 logarithm (lg). The color ranging from blue to red indicates the relative protein abundance from low to high. **(B)** Taxonomic assignment of extracellular hydrolytic enzymes at the SEATS and SS1 station. The yellow circle and green circle indicate the FL fraction and the PA fraction, respectively. The size of the circle indicates the relative abundance of proteins from the taxa.

Table S1. The physicochemical parameters at different depths of stations SEATS and SS1.

Stations	Depth	Temperature (℃)	Oxygen (mg/L)	Salinity (PSU)	Chlorophyll (μg/L)	NO_2^- (μmol/L)	$NO_2^-+NO_3^-$ (μmol/L)	PO_4^{3-} (μmol/L)	SiO_3^{2-} (μmol/L)	[b]POC export flux (mmol C m^{-2} d^{-1})
SEATS	5 m	29.63	5.83	33.71	[a] -	-	-	-	1.674	2.9±0.7
	DCM	21.84	6.01	34.61	0.7514	0.096	3.336	0.250	4.567	
	200 m	13.61	4.43	34.51	-	-	17.557	1.204	25.766	
	750 m	6.02	2.75	34.47	-	-	35.983	2.615	104.326	
	3000 m	2.35	3.55	34.62	-	-	38.885	2.830	147.576	
SS1	5 m	30.22	5.74	33.41	0.0338	-	-	-	2.003	2.0±0.4
	DCM	22.85	6.25	34.48	0.5047	0.122	1.353	0.231	4.670	
	200 m	15.20	4.13	34.56	-	-	16.451	1.079	20.214	
	750 m	6.03	2.69	34.47	-	-	34.777	2.619	105.316	
	3000 m	2.37	3.48	34.62	-	-	38.250	2.834	147.064	

Note: [a] dash line indicates measured values below the detection limit. [b] Data of POC export fluxes at the base of euphotic zone at the SEATS and SS1 stations are from a previous study conducted on the same cruise (25).

Table S2. The relative abundance of seed proteins across KEGG orthology in each sample from stations SEATS (Table S2A) and SS1 (Table S2B) (see excel file).
